# Development of Virtual Reality Scenarios Addressing Gender-Based Violence in Health Sciences Education: Qualitative Approach

**DOI:** 10.2196/76098

**Published:** 2026-03-18

**Authors:** Belén Valverde-Alirangues, Marta Benet, Mar Carrió

**Affiliations:** 1 Health Sciences Education Research Group, Department of Medicine and Life Sciences (MELIS), Universitat Pompeu Fabra, Barcelona, Spain; 2 Sant Joan de Déu Research Foundation, Barcelona, Catalonia, Spain; 3 Department of Optics and Optometry, Faculty of Optics and Optometry, Universitat Politècnica de Catalunya, Carrer del Violinista Vellsola, 37, 08222 Terrassa, Barcelona, Catalonia, 08222, Spain, 34 609834786

**Keywords:** gender-based violence, women’s health, virtual reality, professional education, problem-based learning

## Abstract

**Background:**

Gender-based violence (GBV) is a public health issue affecting 1 in 3 women globally. Its impact on women’s health is challenging, including physical, mental, and social consequences. Health care professionals have a unique opportunity in identifying and supporting GBV survivors, but there is a lack of adequate training.

**Objective:**

This study aims to develop educational resources based on problem-based and experiential learning approaches using virtual reality (VR) scenarios for health sciences students to enhance their skills in addressing GBV.

**Methods:**

A co-creation approach was adopted, encompassing 3 main strategies. First, a focus group was conducted with frontline professionals experienced in GBV. Second, co-creation workshops involved professionals from diverse fields, including higher education pedagogy, gender and public health, nursing and medical education, and immersive technology. Third, expert consultation with frontline professionals ensured coherence between the educational resources and real-world challenges. Following this phase, a first iteration of the materials was piloted with students to assess usability and relevance.

**Results:**

The thematic analysis of the focus group content led to the identification of 9 categories illustrating the competencies and knowledge areas considered relevant to address GBV. As a result of the co-creation workshops, these categories were translated into 18 learning needs, and 4 use cases for the VR component were also identified. The VR scenarios were designed to cover critical GBV situations, fostering transversal skills, such as empathic communication, ethical decision-making, and interdisciplinary collaboration. Two didactic methodologies were proposed for each scenario: a problem-based learning sequence and a single experiential learning session approach, culminating in 4 VR videos and their methodological guides.

**Conclusions:**

The grounding of these educational resources in real-world scenarios, in conjunction with the competencies identified by frontline health and social care professionals with expertise in GBV, ensured alignment with the challenges professionals face in their practice. This helped bridge the gap between theory and practice, offering an innovative approach to GBV education for students of health sciences.

## Introduction

Gender-based violence (GBV) has been identified as a prevalent and globally recognized public health problem, affecting 1 in 3 women and girls during their lifetimes [1]. The term “GBV” is an umbrella concept encompassing all forms of violence perpetrated against women, including physical, psychological, sexual, and other types. These harmful practices occur across various contexts and at different stages of women’s lives. This study seeks to comprehend the phenomenon in its intricacy, which is deeply rooted in the prevailing gender inequality [2]. As intimate partner violence is the most prevalent form described, this study primarily focuses on this type due to its widespread occurrence and relevance in clinical settings. Furthermore, it represents a major concern for women’s health, including not only physical injuries but also numerous mental and social problems for the survivors and their children [2].

There are multiple health problems related to GBV listed, such as sexually transmitted infections, intrauterine bleeding, gastrointestinal problems, neurological disorders, chronic pain, alcohol use, depression, anxiety, and posttraumatic stress disorder, as well as suicide and death by homicide [3]. Moreover, there are psychosocial issues intimately related to GBV survivors that are not rigorously captured within the symptomatology of mental disorders. These often include nonclinical and relational forms of distress associated with poverty, relatively weak legal systems, and protection issues, among others [4].

Therefore, because of its prevalence and high impact on women’s health, addressing GBV must also be closely integrated into health care services, complementing the social care services. Health care services play a crucial role, as many GBV survivors seek care at these facilities without having their specific needs adequately addressed [5]. Health care professionals (HCPs) have a unique opportunity to detect and support survivors by promoting women’s safety and helping them to prevent the worst consequences [6]. Despite acknowledging the importance of health care providers’ capacity to respond effectively to cases of GBV, current scientific evidence highlights the scarcity of HCPs’ abilities and skills to undertake this role [5,7].

In recent years, several educational programs on GBV have been developed for HCPs to improve their competency. Even though recent evidence on this topic suggests positive outcomes, Sprague et al [8] noted that involving professional experts and using specific resources were associated with positive program effectiveness. Numerous interventions have reported an overall positive assessment and significant effectiveness regarding knowledge and ability to address GBV in both health science students and working professionals [9-12]. However, it is challenging to preserve these skills over time [13]. Specific areas of improvement for these interventions have been recognized, such as time constraints, previous acknowledgment, and redundancy in content [14]. Therefore, before proposing any training to professionals, it is essential to determine their learning needs first, considering the sensitivity, complexity, and multifactorial nature of GBV.

Regarding the most suitable educational approach, it is essential to provide opportunities to develop skills in meaningful, real-world scenarios within a safe environment, incorporating active learning methods, such as problem-based learning (PBL), experiential learning, simulation  [15], or immersive approaches. In this regard, a recent review highlights that learning is more effective when active educational strategies are used rather than relying solely on theory-based approaches  [16]. Despite the documented benefits of active learning methods, it is important to acknowledge that they may also elicit a range of emotions and potentially increase stress levels among students as they engage with challenging concepts embedded in complex scenarios with a high emotional burden  [17].

Virtual reality (VR) can contribute to active learning by offering immersive experiences that simulate professional situations, providing a realistic context for learning. The use of VR has been shown to be effective in enhancing learning outcomes while also significantly reducing the psychological pressure often experienced by learners  [18]. Moreover, this technology has proven to be a pedagogically promising approach in terms of both productivity and learner satisfaction  [18,19]. VR scenarios enable the recreation of situations that closely mirror real-life contexts, allowing for the in-depth analysis of subtle elements, such as character personality and attitudes, verbal and nonverbal communication, and contextual details within the scene.

Combining immersive VR scenarios with other active learning methodologies, such as PBL and experiential learning (EL), has the potential to enhance the learning process. In the PBL approach, the VR scenario can serve as a case study to initiate an inquiry process that leads to an in-depth exploration of causes, theoretical frameworks, and various approaches described in the literature for addressing cases of GBV. Traditionally, the PBL methodology involves a sequence of small-group tutorial sessions, concluding with a synthesis session in which the case findings are discussed and strategies for addressing the case are reflected upon [20].

Alternatively, VR scenarios can be integrated into a shorter sequence based on Kolb’s experiential learning model, as revised by Morris  [21], within a single session. This approach is grounded in the reflection and conceptualization of experience, where learning begins with a specific situation in which learners actively participate and which is embedded in a rich context. In this regard, VR serves as a powerful tool to introduce such experiences to students  [22]. This is followed by critical reflective observation, during which learners review the experience, pose questions, challenge actions, and collaboratively analyze the situation  [23]. The process then advances to abstract conceptualization, where new ideas are developed or existing ones refined  [21].

Despite the numerous educational interventions described in the existing literature [24] and the encouraging outcomes frequently reported, uncertainty remains regarding what works and why. This is largely due to the heterogeneity in methods, duration, and outcome measures used across studies  [25].

This study aims to develop innovative educational resources that combine active learning methodologies—including VR scenarios—to enhance the competencies of health sciences students in addressing GBV. The paper presents the co-creation process underlying the design of these resources, which involved identifying the training needs of health professionals, selecting the most relevant cases to be represented in VR, and developing both the narrative scripts and didactic guidelines to support their pedagogical integration. The following sections describe this process in detail and discuss its implications for health education and professional training.

## Methods

### Overview

**Figure 1.**
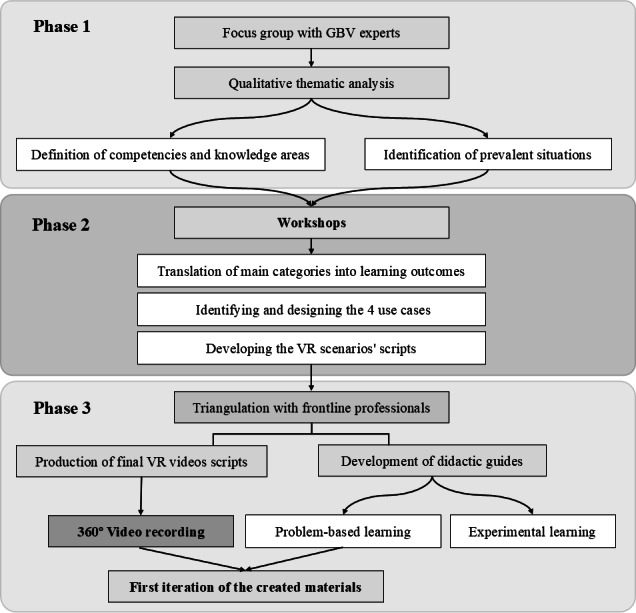
Flowchart illustrating the co-creation process for developing the virtual reality (VR) scenarios. The figure outlines the stages through which the VR scenarios were conceptualized and validated by consulting the original experts, before ultimately being produced. GBV: gender-based violence.

The development of the educational resources was grounded in a co-creation approach with the active involvement of frontline health and social care professionals trained and highly experienced in GBV, higher education pedagogues, lecturers, and people with expertise in immersive technologies throughout all stages of the design process. The co-creation approach included 3 main strategies: focus group, co-creation workshops, and iterative feedback from frontline professionals. This approach was adopted to foster relevance, contextual accuracy, and practical applicability of the final materials. The overall study design was organized into 3 sequential phases, depicted in Figure 1.

The first phase consisted of a focus group to capture the day-to-day practices of the frontline professionals trained and highly experienced in GBV who take care of women who are or have been suffering from GBV. It included professionals with different profiles and was held with two main aims: (1) to establish the competencies and knowledge areas required for HCPs to address GBV and (2) to identify the key situations encountered in daily practice.

Following the focus group’s results, the project moves on to its second phase, which consists of a series of co-creation workshops aimed at (1) translating the competencies and knowledge areas into learning outcomes, (2) identifying 4 use cases to cover the identified learning outcomes, and (3) developing the VR scenarios and the PBL and EL sequences.

Once the 4 use cases were established, the learning outcomes for each case were identified and used as a reference for designing the VR scenarios. This process involved scriptwriting, which included composing the scripts with dialogue, actions, and interactive elements. Additionally, the development of the characters featured in the scenarios was carried out, with key aspects of their physical and behavioral attributes defined to enhance intersectionality by incorporating additional axes of inequality, such as ethnicity, profession, migrant status, and age. To complete this second phase, a storyboard was created to visually represent the sequence of scenes. For the 2 distinct methodologies selected to integrate the VR scenarios into educational practice—PBL and EL—an initial draft of the corresponding sequences was developed alongside the design of the VR scenarios.

In the third phase, the VR scenarios underwent expert validation by frontline experts, who were the professionals from the initial focus group. This process was undertaken to ensure rigor in terms of real-world applicability and relevance. Following the incorporation of their feedback, the final versions of the VR scenario scripts were meticulously refined to ensure maximum reliability, culminating in the recording of the scenes. Subsequently, teaching guides were developed based on the previously drafted PBL and EL sequences to facilitate and standardize the implementation of the educational resources. These guides provide a detailed description of both the pedagogical foundations for classroom application and the theoretical framework required for HCPs to address GBV. Their purpose is to serve as training materials for educators seeking to incorporate these resources into their teaching practice. Finally, a first iteration of the materials was developed to explore its efficacy and areas of improvement.

### Phase 1: Exploration and Identification of Learning Needs and Use Cases

#### Focus Group Participants

Participants for the focus group were selected through intentional sampling, applying both homogeneity (fulfilled by all participants) and heterogeneity (to ensure diversity within the sample) criteria (Table 1). The homogeneity criterion required participants to be trained and highly experienced in GBV and to be currently working as frontline professionals in health or social care. The heterogeneity criteria included level and type of service (primary health care, hospital care, essential and specialized social services, community services for women), age (younger or older than 40 years), gender (female, male, and nonbinary), geographical area (urban or rural), training in cultural diversity, and involvement in GBV coordination bodies. The intentional sampling aimed to maximize diversity across these dimensions to ensure a broad range of perspectives.

**Table 1.** Distribution of the focus group participants according to their profiles, indicating the criteria used to assess homogeneity and heterogeneity among groups.

Participant code	Health care role								Sociodemographic axis						
	Primary health care			Hospital health care	Social services				Age (y)		Gender			Geographical area	
	Doctor or physician	Social worker	Expert on GBV^a^	Midwife	Basic	Social emergency services	Hosting services	Attention services for women	<40	>40	Female	Male	Nonbinary	Rural	Urban
P1			✓					✓		✓	✓			✓	
P2	✓		✓							✓	✓				✓
P3			✓	✓					✓		✓			✓	
P4		✓	✓		✓				✓		✓				✓
P5			✓			✓	✓			✓	✓				✓
P6		✓	✓		✓					✓	✓				✓

a GBV: gender-based violence.

The rationale for selecting highly experienced frontline professionals was based on the assumption that those with extensive experience in addressing GBV are best positioned to identify the competencies and knowledge areas required in daily practice. In contrast, professionals with limited experience may have a less comprehensive understanding of the skills needed to effectively manage situations they have not yet mastered. The selected participants represented a variety of organizations—including health care, social services, and community-based entities—and none were affiliated with the researchers’ institutions, nor did they maintain any previous professional or personal relationship with the researchers.

Six people were selected and contacted by email, all of whom were women. The group included 2 HCPs (a doctor and a midwife), 2 social workers (one specialized in adult and child welfare, the other in addiction rehabilitation), 1 institutionally linked to the women’s affairs organization, and, finally, the last participant belonged to a community-based organization that supports women affected by human trafficking.

#### Data Collection and Analysis

The focus group was conducted online using a semistructured script () that outlined the key issues to be explored and included a set of guiding questions for the moderator. The main topics addressed were competencies and knowledge areas relevant to addressing GBV, how participants identify GBV situations in their daily work with women, and descriptions of typical cases to inform the design of characters (eg, survivor, perpetrator, relatives, professionals) and scenarios (eg, reasons for seeking services, nature of interactions, and service settings where detection occurs) for the VR simulations.

Two researchers were present during the focus group, which lasted 2 hours; one attended as a moderator, while the other observed. The observer documented observations and emergent themes using a predetermined template, supplementing the planned guide.

A transcription of the session was conducted to convert spoken and visual information into written data. This process used both written notes and video recordings to ensure accuracy and completeness.

The qualitative thematic analysis of the focus group transcription was carried out primarily using an inductive approach, aiming to identify emerging competencies and knowledge areas needed to address GBV without relying on a predefined framework. The analysis was conducted independently by 3 researchers. Each researcher followed a structured process: (1) reading the transcript twice to gain a deep understanding of the content, (2) identifying relevant competencies and knowledge areas and assigning corresponding codes, (3) identifying relevant case examples and creating additional codes, and (4) reviewing the coding to ensure internal consistency.

The coding process was conducted using a Microsoft Word document, and the results were summarized in a comparative table. The findings were then discussed in a group meeting, applying researcher triangulation  [26], a technique shown to reduce potential biases in data interpretation. This triangulation exercise also served to reach consensus on the final definition of categories.

**Figure 2.**
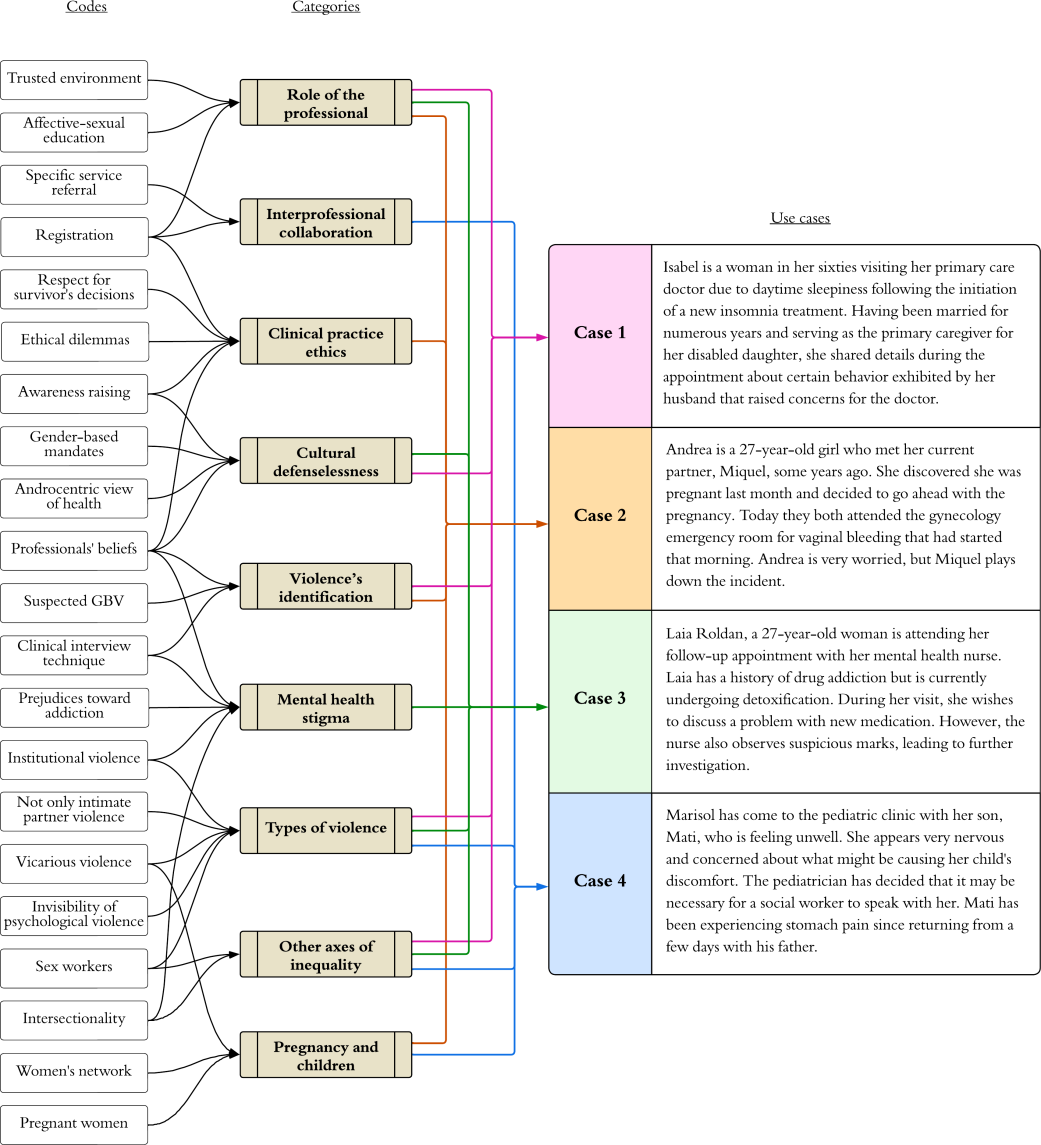
Coding and categorization process. The diagram reflects how the codes and categories that emerged from the co-creation process evolved into the 4 use cases. GBV: gender-based violence.

As a result of this analysis, the most prevalent situations were identified and selected as potential use cases based on the case examples discussed, and 9 main categories of competencies and knowledge areas were defined. Figure 2 describes the categorization process and its application to each case.

### Ethical Considerations

The project was approved by the Institutional Committee for Ethical Review of Projects at Universitat Pompeu Fabra under the approval number 202. The session was recorded with previous explicit and written participant informed consent. Opting for an online environment accommodated transportation and scheduling challenges. Video, alongside audio, was integral to capturing participants’ body language, enriching the analysis and interpretation of their narratives [27]. During the focus group transcription, each participant was anonymized. Participation was voluntary, and no financial compensation or incentives were provided to the participants.

### Phase 2: Workshops: Learning Outcomes and Virtual Reality Scenarios Design

A series of co-creation workshops was carried out with the aim of (1) translating the competencies and knowledge areas into learning outcomes, (2) identifying and designing 4 use cases to address the identified learning outcomes, and (3) developing the VR scenarios along with the PBL and EL sequences.

The working group responsible for analyzing and creating the scenarios comprised a multidisciplinary team of 2 researchers with expertise in gender and health. These researchers contributed their specialized knowledge to understanding GBV and its impact on the health care environment. The group was joined by 2 specialists in health education and an educational innovation technician, whose contribution made it possible to contextualize the findings in relation to health education training and practice. Moreover, the working team included 2 technicians responsible for the production and development of the VR videos linked to the research. These technicians ensured the technical and didactic integration of the audiovisual material in the analysis of the results.

The first workshop served to translate the content of the 9 identified categories into 18 learning outcomes and to define 4 use cases, which would form the basis for the development of the educational resources.

Four additional co-creation sessions focused on designing the use cases, VR scenarios, and the first drafts of the PBL and EL sequences. The second workshop focused on distributing the learning outcomes across the 4 use cases and cross-checking that all were adequately covered. The key aspects of each use case were then defined. The remaining 3 sessions were dedicated to developing the VR scenarios for each use case and the corresponding methodological sequences. In particular, the first drafts of the scripts were produced, with special attention to the dialogues and actions that would best address the specific learning needs. A detailed description of the characters was also created at this stage. Additionally, an initial draft of the key elements for the PBL and EL sequences was developed, including relevant content and frameworks to support the teaching and learning process.

### Phase 3: VR Scenarios’ Recording and Educational Resources Development

To ensure alignment with the real-world situations that professionals face in their daily practice, the resulting scenarios were sent to the frontline professionals who had participated in the focus group. This step was considered a form of contextual validation to ensure appropriateness and relevance. In fact, 1 scenario was significantly revised to better reflect real-life conditions. Initially, the research team had proposed incorporating the stigma surrounding mental health and addictions in 1 use case. However, feedback from experts indicated that this approach might reduce the impact of the case, as instances of sexual exploitation are often closely linked to drug addiction, frequently facilitated by the survivor’s partner. To maintain fidelity to real cases while ensuring accessibility for novice students, the script was accordingly revised to address this concern.

Finally, the scripts for recording the 360° scenarios were produced, and the didactic guides associated with each use case were simultaneously developed to facilitate their future implementation. These guides provide essential pedagogical support for anyone intending to implement the scenarios, offering structured guidance for educators, while both the guides and the accompanying videos are freely accessible, ensuring open access to the resources for broader educational use. Both the guides and the educational intervention rely on 2 didactic methodologies: a classic sequence of PBL sessions and single sessions of EL based on Morris’ cycle [20]. These methodologies are designed to immerse students in real-world scenarios, fostering active engagement and critical thinking. The development process was further refined through an initial iteration of the materials using the classic PBL sequence, ensuring both clarity and pedagogical effectiveness of the resources. This initial iteration will also help identify areas for improvement and refine the use of the scenarios as well as the content of the didactic guides. The test included 64 students enrolled in a degree program in diagnostic imaging and nuclear medicine. A quasi-experimental pre-post design was used, using questionnaires that incorporated the Spanish-translated Perception Scale Partner Violence in Nursing Education (PSPV-NE) scale [28], which evaluates students’ perceptions of GBV, including general training, survivor and aggressor identification, and professional roles and values in addressing the issue. The primary outcome was analyzed using the Student *t* test, with a significance threshold set at an *α* of .05.

## Results

In each phase of the co-creation, certain results have been obtained, which have enabled the next phase to proceed.

### Phase 1: Exploration and Identification of Learning Needs and Use Cases

As a result of the focus group analysis, 9 main categories were generated. In addition, the most common and critical situations of GBV 4 cases were identified: intimate partner violence during pregnancy, multiple types of violence against women in situations of drug addiction, psychological abuse in elderly women, and violence against the mother perpetrated through her children. The coding and categorization process is described in Figure 2.

In addition, there was consensus in the focus group on transversal aptitudes for addressing GBV. The main skills described that workers should have to deal in an appropriate manner with GBV situations were as follows: (1) a professional and careful interaction, empathic but not paternalist (“It is not just a conversation [...]. Words must be chosen carefully” [P3]); (2) establishing a climate of confidence and trust (“Generate trusting environments and relationships—so that they can explain, identify, and not to feel judged” [P1]); (3) preventing revictimization (“Women often arrive blocked, they want oblivion, they don’t want to explain the same thing fifteen times” [P2]); and (4) social care and health care system awareness, knowledge about services, and its limitations (“It is important to confront the difficulty, accept the situation, take care of the victim and tell them that you will transfer them to a place where they can be looked after” [P5]. “It may be that there is a three-month wait for access to a resource, that it is far away [...], or that you do not have access because in your case it is ‘just’ psychological violence” [P4]).

Furthermore, the group underlined the importance of representing and taking into account other axes of inequality faced by the survivor and the professionals’ willingness to set aside their own prejudices on the subject, in order to address the situation holistically.

Professionals have a lot of prejudices about these women, about their lives, about their potential to get out of the situation.

[P6]

There is structural violence and many culturally normalized behaviors [...]. There is not only intimate partner violence, other groups such as LGBTI community, sex workers or mental health issues must also be considered.

[P5]

Finally, a key point that emerged from the session was respect for the bioethical principle of autonomy and its dichotomy with respect to nonmaleficence. For professionals, this is a problematic issue, and they describe a tendency to solve it in the quickest possible way, sometimes reproducing patriarchal logic. However, it is not only key to respect women’s decisions (protecting their safety) but also their time in the recovery process from violence.

It is important to avoid insisting on the denunciation as if everything ended with it [...] a very complicated process of reparation begins, which often starts with a psycho-emotional blockage.

[P5]

Health professionals have the tendency to tell others what to feel or do, but ultimately it is the individual who has to decide.

[P2]

Do not want to be women’s saviors—they are the leading characters, and they are the ones who have to play an active role.

[P3]

### Phase 2: Workshops: Learning Outcomes and Virtual Reality Scenarios Design

Based on the main categories that emerged in the focus group, 4 different VR scenarios have been created, as well as the learning outcomes related to each case. It was because of their frequency and didactic potential that these 4 key cases were selected.

#### Case 1: Doctor, These Pills Make Me Sleepy

The main topics related to this case were cultural defenselessness to GBV and effective and empathic communication (Multimedia Appendix 1). It shows psychological violence toward an elderly woman. Thereafter, specific learning outcomes were assigned according to the starting topics: (1) developing interview technique, active listening, and empathy; (2) applying different communicative and relational strategies based on the survivor’s situation; (3) recognizing individual value systems and fostering a critical examination of these constructs; and (4) identifying GBV as a determinant of women’s health.

#### Case 2: Pregnant Women’s Spotting

In this case, principal topics related were clinical practice ethics, pregnancy as a vulnerability scenario, and HCP assessment and intervention (Multimedia Appendix 1). It shows both physical and sexual violence perpetrated by the intimate partner on a pregnant woman. Particularly in this case, 2 versions of the ending are available, depending on a student’s decision, which will confirm or maintain the doubt that her situation is one of GBV. The specific related learning outcomes are (1) reflecting on ethical dilemmas arising when GBV is suspected and how to intervene as a professional; (2) developing an awareness and comprehensive understanding of the vulnerable circumstances faced by pregnant women, and acquiring the necessary skills to respond appropriately to their needs; (3) applying risk evaluation tools for women experiencing GBV; and (4) constructing a decision-making framework that incorporates complexity, consistently placing the needs of the woman at the forefront.

#### Case 3: Laia, Have You Returned to Drug Use?

This third case is associated with professionals’ biases and drug addiction as a vulnerability scenario (Multimedia Appendix 1). It reveals sexual and institutional violence toward the survivor. The related learning outcomes are (1) recognizing indicators of GBV in complex contexts, including addiction scenarios, and implementing an appropriate intervention; (2) identifying stereotypes and beliefs that may impact HCP practices through the application of critical thinking and reflective practice; and (3) elucidating the concept of institutional violence and secondary revictimization, and discerning its manifestation within professional practices.

#### Case 4: Mati Has a Bellyache

Finally, the last case created is based on multidisciplinary collaboration and the inclusion of children as a vulnerability scenario (Multimedia Appendix 1). The last specific learning outcomes elaborated were (1) understanding the protocols for responding to situations of suspected GBV, particularly in cases involving minors; (2) identifying an instance of vicarious violence through the narrative and exploration of a child or their mother; and (3) recognizing the importance of documenting aspects involving GBV in the medical record and its advantages and disadvantages.

### Phase 3: VR Scenarios’ Recording and Educational Resources Development

The feedback received regarding the scripts following the expert consultation process was, in general, validating. The frontline professionals highlighted both the similarity of the cases to current clinical practice, as well as the importance of the learning outcomes proposed by the researchers. Finally, the scenarios outlined above were recorded using a 360° recording camera. Learning guides were also drafted for each case based on the learning outcomes obtained.

**Figure 3.**
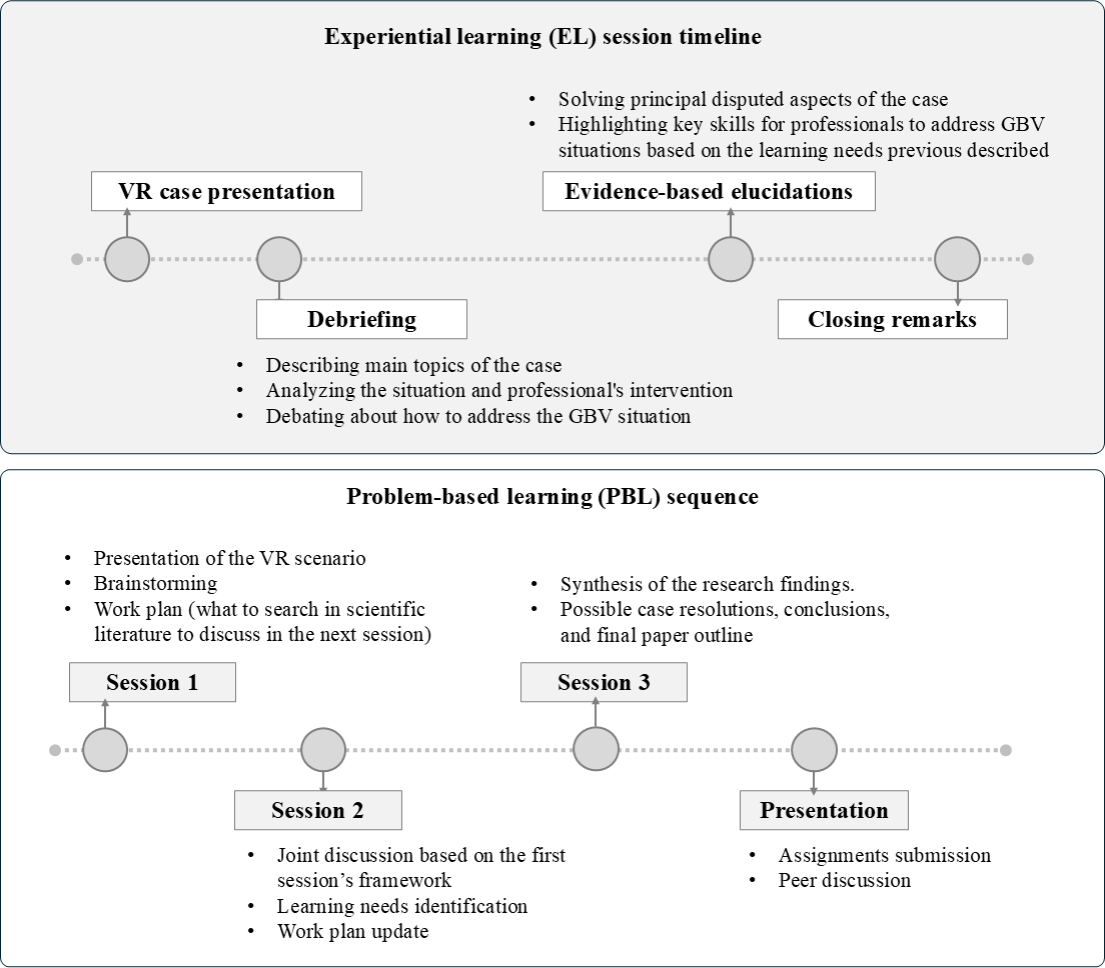
Proposed learning sequence for the implementation of virtual reality (VR) scenarios, using experiential learning teaching methodologies with a single 2-hour session and problem-based learning with four 2-hour sessions. GBV: gender-based violence.

Two didactic methodologies were proposed for their application (Figure 3), depending on the objectives and logistical needs of the educational centers. For each of them, a didactic support guide was prepared containing the learning outcomes of each case, the scripts with the most noteworthy elements, a theoretical section concerning the essential concepts of the case, and finally, a list of useful resources available [29,30]. These guides will prove valuable for teachers who apply these interventions, not only in terms of content resources but also to unify criteria in the session’s methodology and evaluation. Both the teaching guides and the 360° videos are available in open access format.

The first methodology proposed was a PBL classical sequence. By integrating problem-solving methodology, students are encouraged to apply theoretical knowledge to practical situations, going deeper into the subject and skill development in addressing GBV within health care settings. The PBL sequence typically begins with the presentation of a case scenario for detailed analysis. In this study, VR was used as an alternative to traditional written cases. This technological innovation enhances student engagement by providing an immersive, first-hand experience of the situation, thereby creating a strong foundation for knowledge construction. The PBL method further reinforces content through multiple sessions, each lasting approximately 2 hours and conducted in small groups. These sessions facilitate in-depth case analysis and encourage students to reach the highest levels of Bloom’s taxonomy, including the creation of novel products and the generation of alternative solutions [31]. The Bloom framework is particularly relevant in PBL contexts, as it informs the design of complex, real-world problems that require learners to use higher-order cognitive skills.

A single session of approximately 2 hours using EL was also proposed. The EL session is grounded in concrete reflection on the experience, providing a highly valuable didactic opportunity despite limited theoretical exploration. This approach allows students to engage deeply with the scenarios, fostering the specific learning outcomes through an extensive peer debriefing. This methodology is key to theorize and conceptualize professional activity based on a profound reflection of experience and to be able to analyze all the contextual elements of each scenario and its transferability to other contexts. This debriefing comprises a major part within the session, not only describing and analyzing what has been observed in the case but also culminating with a theory overview of relevant issues to address any doubts and concerns that have been raised in the debriefing.

Regarding the explorative iteration of the materials, the mean scores obtained on the PSPV-NE assessment were higher across all items following the intervention, reaching statistical significance (*P*<.05). Analysis of the 4 subscales similarly revealed significant improvements after the educational training, as did the participants’ ability to correctly identify both the survivor and the perpetrator. However, knowledge regarding the role of HCPs in addressing GBV, while showing a positive trend, did not reach statistical significance. These findings highlighted the need to reinforce the role of HCPs in addressing GBV within the educational materials, which was subsequently incorporated into the instructional guides.

## Discussion

This study describes the design of VR-based educational materials developed through a co-creation methodology and discusses how this approach enhances the clinical relevance, realism, and educational alignment of GBV training in health sciences curricula. The collaborative involvement of HCPs, GBV experts, and specialists in educational innovation and immersive technologies resulted in the development of four immersive VR scenarios representing frequent and complex clinical situations: psychological violence within an elderly married couple, physical violence during pregnancy, GBV in a woman with substance use disorder, and vicarious violence. These scenarios were complemented by comprehensive teaching guides, enabling their direct integration into health sciences curricula and facilitating their use by university instructors.

The involvement of frontline HCPs with extensive experience in GBV was essential for identifying both the most pressing training needs of HCPs and the types of cases most commonly encountered in routine clinical practice. Their contributions informed the definition of 9 key categories that guided the selection of the 4 use cases and the identification of transversal competencies required for effective clinical responses to GBV. This methodological approach constitutes a significant strength of the study in terms of applicability and reliability. As noted by Van Weel-Baumgarten et al [32], co-creation processes enhance relevance and feasibility when experts are actively engaged throughout the design process.

The integration of GBV experts alongside academic and technical profiles further strengthened the educational alignment and implementation potential of the training program. This interdisciplinary collaboration is consistent with the recommendations of Surapaneni [33], who emphasized the importance of expert collaboration in the development of complex educational interventions. The use of focus groups facilitated in-depth and reflective dialogue [34], enabling the identification of critical professional skills, including empathic yet nonpaternalistic communication, the establishment of trust, prevention of revictimization, and awareness of health care and social system limitations. These competencies were explicitly embedded within the learning outcomes of each VR scenario, ensuring that the educational resources accurately reflect real-world clinical experiences.

In line with the work of Jiménez-Rodríguez et al [35], the use of videos simulating clinical scenarios may support a more effective alignment between theoretical knowledge and practical application, particularly when scenarios incorporate the ethical, emotional, and relational complexities identified by professionals themselves. While numerous educational initiatives addressing GBV have been described in the literature, substantial curricular limitations persist [5]. This study contributes to addressing these gaps by translating professional expertise into immersive VR scenarios that go beyond identification, incorporating ethical dilemmas, professional biases, and intersecting vulnerabilities such as pregnancy, addiction, older age, and vicarious violence.

Previous research has shown that educational interventions can improve knowledge, attitudes, skills, and behaviors related to GBV [36], with simulation-based and experiential learning approaches offering additional benefits [37]. The findings of Adánez-Martínez et al [38] further support the educational value of simulation and problem-solving videos, reinforcing the suitability of VR for representing complex and sensitive GBV cases. In this study, the pilot implementation indicated a positive educational potential, as reflected by increased mean PSPV-NE scores across all subscales and improvements in students’ ability to identify both survivors and perpetrators of GBV.

These findings align with prior evidence suggesting that immersive VR experiences can promote behavioral change in educational contexts [39], particularly when combined with structured reflection and debriefing [40]. Although improvements in knowledge regarding the specific role of HCPs did not reach statistical significance, this result was used formatively to refine the instructional guides, placing greater emphasis on professional responsibility and system-level intervention. This iterative refinement responds to ongoing challenges in GBV education related to the use of robust evaluation strategies [5,41].

Beyond methodological innovation, this intervention also addresses content-related gaps in existing curricula. As noted by previous authors, medical education often prioritizes risk factors and the identification of GBV [42]. In contrast, this study integrates ethical considerations, institutional violence, and broader axes of inequality that shape women’s health care experiences, reflecting the themes raised by expert participants. While the study makes a meaningful contribution to GBV education, further research using more robust and longitudinal evaluation designs is warranted.

From a practical standpoint, the open-access availability of the 360° VR videos and accompanying teaching guides supports dissemination and scalability across educational contexts. Overall, the educational resources developed represent a valuable contribution to the integration of GBV education into health sciences curricula. By grounding VR scenario design in professional expertise and combining immersive technology with active learning methodologies, this intervention offers a safe, experiential environment for addressing complex and sensitive clinical situations. The co-creation process presented may therefore serve as a transferable model for the development of educational interventions in other complex areas of health care education.

## Supplementary material

10.2196/76098 Multimedia Appendix 1 Virtual reality scenarios and instructional guides for addressing gender-based violence in health sciences education.

## Abbreviations

EL: experiential learning
GBV: gender-based violence
HCP: health care professional
PBL: problem-based learning
PSPV-NE: Perception Scale Partner Violence in Nursing Education
VR: virtual reality
