# Supplementary material for: Development of Virtual Reality Scenarios Addressing Gender-Based Violence in Health Sciences Education: Qualitative Approach
Source: JMIR Med Educ. 2026 Mar 18;12:e76098. doi: 10.2196/76098 (PMC12998608; doi:10.2196/76098)
Supplement: Multimedia Appendix 1 [file mededu-v12-e76098-s001.pdf]

Discussion group leader

Presentation of the discussion group

1. Greetings and introduction of the moderator and observer
2. General information on the topic and objective of the session
    a. In reference to the call for participation...
    b. We are conducting a study on...
3. Presentation of the selection criteria and common aspects
    a. All of you are carriers who work at AP...
    b. The information shared in this discussion group is completely confidential...
4. Rules of operation
    a. Questions will be asked about...
    b. You can express yourselves freely and respect each opinion; each person has their own view of reality
    c. It is just as important to speak as it is to listen to what other people have to say, so we will try not to all speak at once
    d. As mentioned, the session will be recorded for research purposes... Are there any objections?
5. Introduction of participants

Question guide - discussion group

In order to develop the guide, we need to be clear about the following aspects:

1. General and specific educational objectives:

[Based on what we have discussed in the meetings, we want the professionals themselves to set the most relevant educational objectives to be implemented in the experience, so we can ask them...]

- *What do you think is most important or a priority when it comes to equipping future professionals with the tools that will enable them to correctly detect possible cases of gender-based violence?*

- *What training would you have liked to receive during your initial training? (Sometimes taking a personal approach can reveal other perspectives).*

2. Detection indicators

[We want to know these indicators because we will reproduce them in the simulation, and in this way the student will learn to identify them]

- *How do you carry out detection? What is important to take into account when detecting a situation of violence? What should you do when a woman tells you about or gives you signs of a situation of violence?*

- *What are the signs of gender-based violence that you encounter? What makes you suspect a situation of male violence?*
- *Do these signs vary depending on the profile of the women?*
- *What type of violence do you consider to be the most prevalent? Is it also the most detected? (If the answer is no) Why?*
- *What types of cases or situations do you consider most relevant when training future students? (Due to their novelty, their prevalence, their impact on the victim's life)*
- *What strategy or steps do you follow when you suspect a situation of violence?*
- *Once the case is confirmed, how do you proceed?*

Spaces: We will have to ask, or decide ourselves based on what we learn in the discussion groups, where the scene will take place.

Emergency room? Family doctor's office? Traumatology? Gynaecology?

3. Characters:

[These questions are aimed at creating a good psychological profile of the characters that appear on stage]

EXPERT PROFESSIONAL CHARACTER:

- *On an emotional level, how do you feel when you encounter a case?*
- *How does it affect you emotionally and professionally that your workplace specialises in gender-based violence?*
- *In relation to cases or possible situations of male violence, what are the hardest moments of your work, the ones that carry the most emotional weight?*
- *Do you feel that it is an individual task, or do you feel supported by a team of people when carrying out your tasks?*

CHARACTER OF THE AGGRESSOR

- *What signs make you think that the partner is the aggressor?*
- *When the abuser is the partner, how do you proceed?*

- *What are the signs of gender-based violence that can be identified by analysing the relationship between the patient and their partner (aggressor)?*

THE ROLE OF THE FAMILY

- *What role do accompanying family members (mothers/fathers, siblings, children) play in identifying the situation during the consultation?*

PERSONALITY OF THE VICTIM OF VIOLENCE

We understand that we cannot ask what the profile of a victim of gender violence is, because we are all susceptible to being one, and because it is not true that there is a specific profile for either the victim or the aggressor (age, ideology, ethnicity, etc.). Furthermore, we are all different, each with our own experiences and emotions, and we cannot generalise emotions or cases. However, for the script, we need to gather as much information as possible about the wide range of profiles we encounter.

The psychological profile and emotions of these women will have common traits, but they will also differ in other aspects. For scripting, we must identify both the common and particular traits and emotions. These aspects **can best be identified with the help of an expert professional.**

We will need to identify emotions and feelings:

- *Feelings and emotions at each stage or moment of the process. Guilt, confusion, denial, frustration, anger, fear, insecurity, empowerment...? What are these emotions due to?*
- *Do they feel safe in the healthcare setting that is treating them? Does the healthcare setting have advantages or strengths when it comes to dealing with cases, compared to a police or court setting? If so, how can these be exploited to make the victim feel comfortable and ensure that the process runs smoothly?*
- *Difficulties and frustrations: institutionalisation, bureaucracy, etc.*
- *Are victims of gender-based violence aware of the law, their rights, protection and protocols? Are they aware of the services available?*
- *If I don't know them, what is the best way to communicate this assertively, while accompanying them?*

Conclusion

Express gratitude and explain how their participation will contribute to the study.

Discussion group form

<table>
<tr><td>Date:</td><td rowspan="6">List of focus group participants:</td></tr>
<tr><td>Location:</td></tr>
<tr><td>Duration:</td></tr>
<tr><td>Recording: Yes / No</td></tr>
<tr><td>Moderator</td></tr>
<tr><td>Observer</td></tr>
<tr><td colspan="2">Objective:</td></tr>
<tr><td colspan="2">Brief summary of the focus group</td></tr>
<tr><td colspan="2">List of topics discussed:</td></tr>
<tr><td colspan="2">Relevant observations on how the focus group went:</td></tr>
</table>

Discussion group observation sheet

General observations

Participants' observations

Time	Participant	Observation

Final comments from the observer

